# Hepatic Encephalopathy Caused by Long-Term Amiodarone Use

**DOI:** 10.7759/cureus.50690

**Published:** 2023-12-17

**Authors:** Naoki Murata, Toshinori Nishizawa, Yuntae Kim, Hiroko Arioka

**Affiliations:** 1 Department of General Internal Medicine, St. Luke’s International Hospital, Tokyo, JPN; 2 Department of General Internal Medicine, St. Luke's International Hospital, Tokyo, JPN; 3 Department of Gastroenterology, St. Luke’s International Hospital, Tokyo, JPN

**Keywords:** drug-induced hepatitis, severe hepatic encephalopathy and chronic liver disease, non-alcoholic steatohepatitis, serum ammonia, fatty liver disease

## Abstract

This case describes a 72-year-old Japanese woman with hypertrophic cardiomyopathy and non-sustained ventricular tachycardia who had received a total of 215 g of amiodarone over six years and presented with hepatic encephalopathy. The abdominal non-contrast computed tomography showed diffusely increased attenuation of the liver parenchyma. The liver biopsy revealed drug-induced steatohepatitis. No genetic variations in the urea cycle were found. She was ultimately diagnosed with drug-induced steatohepatitis and urea cycle abnormalities caused by long-term amiodarone use. Amiodarone may cause drug-induced steatohepatitis and urea cycle abnormalities, which could induce hyperammonemia. Although case reports of amiodarone-induced hyperammonemia and hepatic encephalopathy have already been reported, we present a typical picture of an amiodarone-induced bright liver, including the mechanism of amiodarone-induced hyperammonemia, to provide an educational learning point for many readers.

## Introduction

Amiodarone is the most commonly administered antiarrhythmic drug but shows liver toxicity [1]. Amiodarone accumulates in hepatocytes and affects the pathway of β-oxidation, which causes steatohepatitis-like liver damage and consequently induces liver cirrhosis [2]. In addition, amiodarone impairs the urea cycle by several mechanisms [3]. Thus, hyperammonemia may be caused by steatohepatitis and urea cycle abnormalities due to the long-term use of amiodarone.

A 72-year-old Japanese woman with a history of hypertrophic cardiomyopathy, non-sustained ventricular tachycardia, and long-term amiodarone use presented with disorientation. Her blood tests show elevated ammonia, total bilirubin, direct bilirubin, aspartate aminotransferase, and alanine aminotransferase levels, which were consistent with amiodarone-induced hepatotoxicity given her history of long-term amiodarone use. Although case reports of amiodarone-induced hyperammonemia and hepatic encephalopathy have already been reported [3], we present a typical picture of an amiodarone-induced bright liver, including the mechanism of amiodarone-induced hyperammonemia, which might be an educational learning point for many readers.

## Case presentation

A 72-year-old Japanese woman weighing 45 kg and with a body mass index of 16.5 kg/m2 was admitted to our hospital because of disorientation. Two years before the current presentation, the patient had first experienced seizures and impaired consciousness. These symptoms were considered convulsive seizures and post-ictal states due to influenza virus infection based on the positive test results. The condition was resolved with the initiation of levetiracetam. One year before the current presentation, she developed myoclonus while hospitalized for a right femoral neck fracture in the orthopedic surgery department. This also resolved spontaneously. Until two months before the current presentation, the patient had been in her usual state of health. One month before the current presentation, the patient had been admitted to the local hospital with a fever and chills. She was diagnosed with *Streptococcus agalactiae* bacteremia, treated with cefazolin for two weeks, and subsequently transferred to a rehabilitation facility after three weeks. During her stay at the rehabilitation facility, her dietary intake was insufficient due to her selective eating habits. Three days before the presentation, she became somnolent and was transferred to our hospital for a comprehensive evaluation.

The patient had a 16-year history of hypertrophic cardiomyopathy and non-sustained ventricular tachycardia. An implantable cardiovascular defibrillator had been introduced eight years before, and she had taken a total of 215 g of amiodarone over six years (most recently 200 mg per day). She had never smoked and did not drink alcohol.

On examination, she was disoriented, with a Glasgow Coma Scale of 5 (E3, V1, M1), a body temperature of 37.3°C, a heart rate of 69 beats per minute, a blood pressure of 119/82 mmHg, a respiratory rate of 14 breaths per minute, and an oxygen saturation of 99% while breathing ambient air. Physical examination showed icteric sclera, myoclonic movements of the head and upper limbs, and roving eye movements. She had no liver or spleen enlargement.

Laboratory tests revealed an ammonia level of 246 μg/dL, an aspartate aminotransferase level of 78 U/L, and an alanine aminotransferase level of 60 U/L. Other laboratory tests conducted upon admission and during the two years leading up to admission are shown in Tables 1-2. Results of antibody-based screening tests for hepatitis B, hepatitis C, the human immunodeficiency virus, and syphilis were negative. The level of antinuclear antibody was normal.

**Table 1:** Laboratory data N/A: not assessed

Labs	Two years before admission	One year before admission	Two months before admission	On admission	On discharge (43 days after admission)	Six months after discharge	One year after admission	Reference value
White blood cell count	3600	4300	2900	4000	3700	3300	3600	3300-8600 cells/mm³
Hemoglobin	15.6	11.0	12.3	16.0	12.4	12.1	13.2	11.6-14.8 g/dL
Platelet count	93000	160000	97000	81000	131000	105000	97000	158000-348000 cells/cm³
Total protein	7.4	5.2	5.3	6.6	N/A	6.1	6.4	6.6-8.1 g/dL
Albumin	3.7	2.3	2.6	2.6	2.1	2.7	2.9	4.1-5.1 g/dL
Blood urea nitrogen	17.3	24.4	14.8	34.0	12.2	15.2	14.5	8-20 mg/dL
Serum creatinine	0.75	0.68	0.68	0.85	0.56	0.44	0.46	0.46-0.79 mg/dL
Serum sodium	140	138	143	164	134	142	141	138-145 mEq/L
Potassium	3.9	4.2	4.0	3.9	4.6	3.9	4.0	3.6-4.8 mEq/L
Bicarbonate	N/A	N/A	30.4	32.5	N/A	N/A	N/A	22-26 mmol/L
Total bilirubin	2.3	3.5	2.4	3.4	1.4	1.9	2.0	0.4-1.5 mg/dL
Direct bilirubin	N/A	1.6	0.9	1.2	N/A	N/A	N/A	<0.4 mg/dL
Aspartate aminotransferase	36	40	29	78	30	22	20	13-30 U/L
Alanine aminotransferase	21	42	22	60	25	15	12	7-23 U/L
Gamma-glutamyl transpeptidase	15	34	36	31	29	20	15	<32 U/L
Alkaline phosphatase	250	N/A	68	104	N/A	103	104	38-113 U/L
Ammonia	137	104	N/A	246	72	106	230	0-51 μg/dL
C-reactive protein	1.05	12.8	N/A	1.13	0.28	0.28	0.26	<0.3 mg/dL
Glucose	118	106	103	88	N/A	N/A	N/A	73-109 mg/dL
Amiodarone	1445	1740	1179	998.3	691.7	N/A	N/A	500-1000 ng/mL (200 mg/day, steady state, trough values)

**Table 2:** Other laboratory data Fisher's ratio was calculated by dividing the sum of the concentrations of branched-chain amino acids by the sum of the concentrations of aromatic amino acids. The branched-chain amino acids include leucine, isoleucine, and valine. The aromatic amino acids are tyrosine and phenylalanine.

Labs	Results on admission	Reference value
Zinc	47	80-130 μg/dL
Free carnitine	50.5	36-74 μmol/L
Total carnitine	60.4	45-91 μmol/L
Free T4	1.53	1.0-1.64 ng/dL
TSH	3.66	0.45-4.95 μIU/mL
Serum cholinesterase	139	201-421 U/L
M2BPGi	4.32	<1.00 COI
Type IV collagen 7s	6.8	<4.4 ng/mL
Serum citrulline	60.2	17.9-48.0 nmol/mL
Arginine	88.3	31.8-149.5 nmol/mL
Threonine	93.4	74.2-216.1 nmol/mL
Serine	78.3	91.5-186.4 nmol/mL
Fisher’s ratio	0.9	2.2-4.3

**Figure 1:**
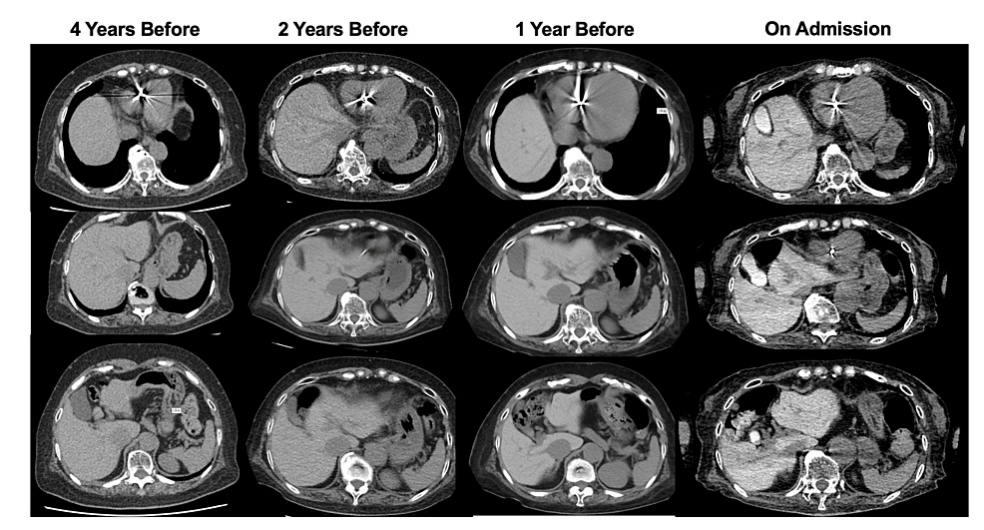
Abdominal non-contrasted CT The non-contrast CT scan of the abdomen at the time of admission revealed diffuse hyperattenuation within the liver parenchyma and the bile in the gallbladder. There was a noted gradual increase in the liver's attenuation when compared to previous CT scans. The mean liver attenuation values were 70 Hounsfield Units (HU) four years before, 81 HU two years before, 99 HU one year before, and 134 HU at the time of admission.

**Figure 2:**
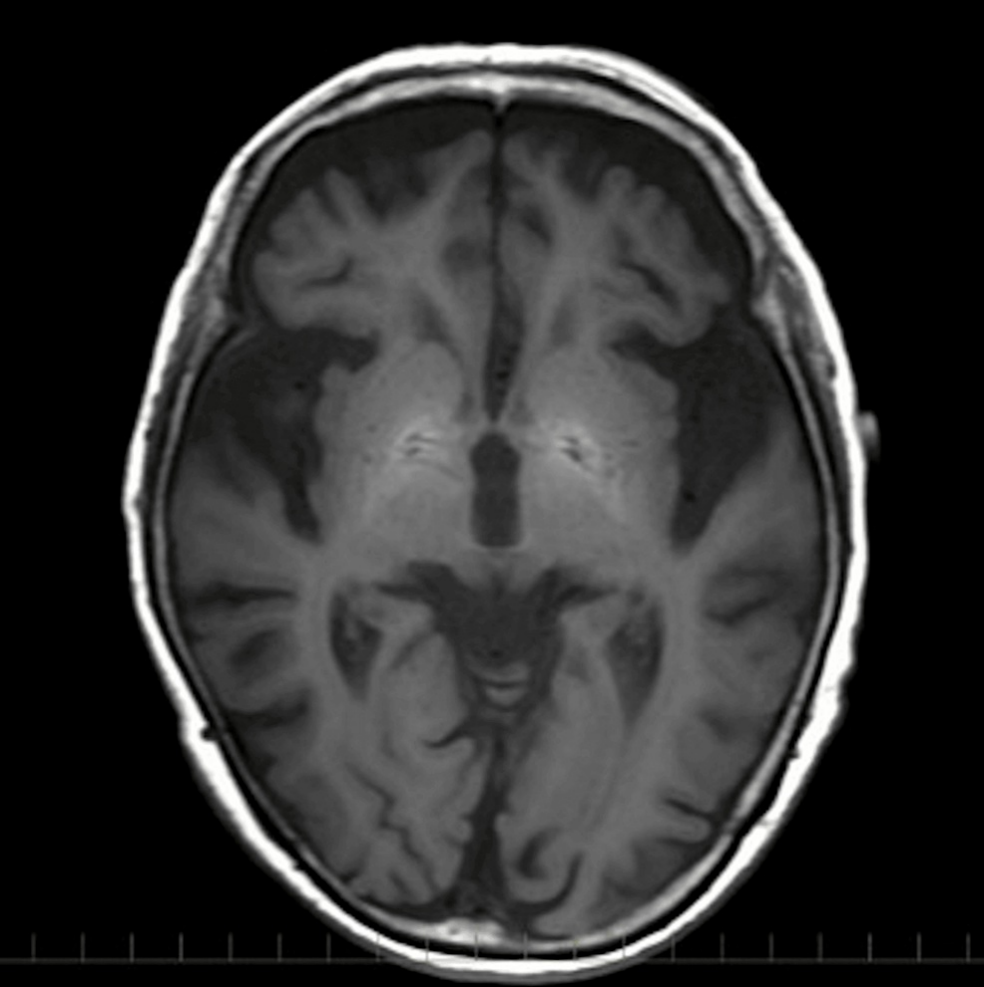
Axial T1-weighted MRI Axial T1-weighted MRI showed a markedly high-intensity area in the globus pallidum.

**Figure 3:**
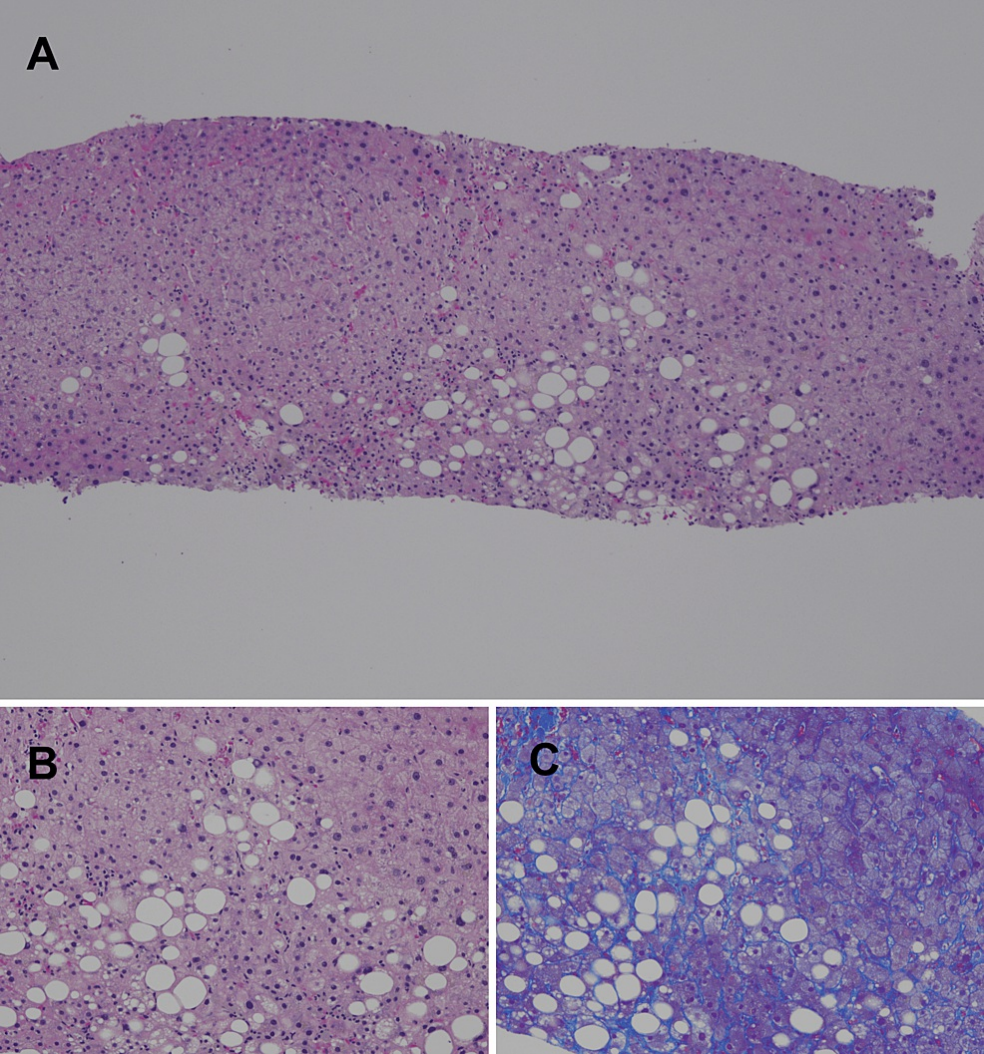
Pathology images of the liver biopsy The hepatic lobule's basic architecture was preserved. Within the lobule, large intrahepatic lipid droplets were present in approximately 20% of hepatocytes. Ballooning of hepatocytes was noted around the central vein. A mild to moderate necroinflammatory response was evident, characterized by scattered pigmented histiocytes. The portal areas showed mild fibrosis and dilation, with a mild lymphocytic inflammatory infiltrate. These findings were observed under hematoxylin and eosin staining at low magnification (A) and high magnification (B), and Masson's trichrome staining at high magnification (C).

Abdominal non-contrast CT showed diffusely increased attenuation of the liver parenchyma and the bile in the gallbladder (Figure 1). Brain axial T1-weighted MRI showed a markedly high-intensity area in the globus pallidum (Figure 2). Electroencephalography showed no clear seizure waves. Contrast-enhanced CT revealed no intrahepatic portosystemic venous shunts. The liver biopsy revealed moderate steatosis with necroinflammatory changes, ballooning degeneration, and pericellular fibrosis, all consistent with drug-induced steatohepatitis (Brunt criteria: grade 2 and stage 2, Matteoni’s classification: type 4, Figure 3). In addition, we examined the exons and intron/exon boundaries of the five key urea enzymes, including ornithine transcarbamylase (OTC), carbamoyl-phosphate synthetase 1 (CPS1), arginase 1 (ARG1), argininosuccinate synthetase (ASS1), argininosuccinate lyase (ASL), N-acetylglutamate synthase (NAGS), and two solute carrier transporter genes (SLC25A13, SLC25A15). However, no genetic variations in the urea cycle were found.

These findings were consistent with amiodarone-induced hepatotoxicity, given her history of long-term amiodarone use. An abdominal CT four years ago revealed normal liver attenuation. However, CT performed two years ago and one year ago indicated a gradual increase in the liver's attenuation. At the time of admission, further diffuse hyperattenuation was observed within the liver parenchyma. In addition, for the two years before the present case, blood tests showed elevated transaminases and ammonia. Therefore, it is plausible that the continued accumulation of amiodarone during this period may have led to drug-induced steatohepatitis. Notably, high-intensity areas in the globus pallidum on T1-weighted MRI suggest chronic liver failure and portal-systemic encephalopathy [4]. Furthermore, hyperammonemia might be induced by not only drug-induced steatohepatitis but also urea cycle abnormalities induced by the long-term use of amiodarone. Genetic variations in the urea cycle were examined [5], but no abnormalities were found. The patient had selective eating habits and showed hyper-citrullinemia along with an elevated threonine/serine ratio. Initially, we considered adult-onset type II citrullinemia as a possible differential diagnosis [6]. However, further investigation revealed no evidence of SLC25A13 gene abnormalities, leading us to conclude that citrullinemia was unlikely in this case. In this case, *Streptococcus agalactiae* bacteremia and dehydration may have contributed to hyperammonemia. Zinc deficiency could also have contributed to the reduced processing of ammonia in the urea cycle, but the degree was mild. Since she had constipation, ammonia production by intestinal bacteria was also considered a contributing factor to hyperammonemia. Although infection, dehydration, zinc deficiency, and constipation may have contributed to hyperammonemia, hyperammonemia has been present despite these improvements. Levetiracetam may pose a risk of hyperammonemia in patients with chronic liver disease, although this risk is lower compared to other anticonvulsants [7]. Chronic liver damage and urea cycle abnormalities were considered more important etiologies in this case. She was finally diagnosed with drug-induced steatohepatitis and urea cycle abnormalities caused by long-term amiodarone use.

On admission, the patient presented with hyperammonemia, which initially improved after fluid administration and discontinuation of amiodarone. However, the ammonia level increased again during hospitalization in the setting of constipation, leading to impaired consciousness. The patient was treated with branched-chain amino acids, rifaximin, carnitine, and lactulose. Thereafter, the ammonia level stabilized below 100 μg/dL, and the patient was discharged on the 43rd day of hospitalization. One year after the cessation of amiodarone, the ammonia level gradually increased regardless of the above treatment; however, there were no further episodes of impaired consciousness.

## Discussion

Amiodarone is a class III antiarrhythmic drug that is used all over the world. The representative adverse effects include toxicities in the liver, thyroid, and lungs. Adverse effects include thyroid toxicity in 1-22%, hepatic toxicity in 15-50%, and pulmonary toxicity in 2-7%. It is recommended that thyroid and liver function be regularly monitored by the laboratory data [8]. Approximately 24% of patients taking amiodarone had asymptomatic elevated serum aminotransferase levels. In various studies, less than 1% of patients reported the development of significant drug-induced liver injury, ranging from symptomatic hepatitis and micronodular cirrhosis to liver failure requiring liver transplantation [9]. Acute hepatocellular injury occurred within 24 hours after the administration of intravenous amiodarone [10]. In addition, due to its long biological half-life of 100 days, the drug's effects may persist for more than three to four months after amiodarone administration is discontinued [11]. Regarding hepatotoxicity, amiodarone accumulates in hepatocytes and affects the pathway of β-oxidation. This leads to apoptosis and necrosis of hepatic cells, causes steatohepatitis, and consequently induces liver cirrhosis [2]. In this case, the liver enzymes gradually improved after the discontinuation of amiodarone.

Amiodarone is recognized to inhibit the urea cycle by the following several mechanisms: First, amiodarone might inhibit carnitine-palmityl transferase 1, which results in a reduced availability of acylcarnitine with impaired beta-oxidation and acetyl-CoA production. Amiodarone also impairs the mitochondrial respiratory chain activity at the level of complexes I and II; therefore, the decrease in acetyl-CoA might reduce the availability of N-acetyl glutamic acid. The reduction of N-acetyl glutamic acid leads to the deterioration of carbamoyl phosphate, which is necessary for synthesizing citrulline in the urea cycle [3]. In this case, hyperammonemia did not improve even one year after stopping amiodarone. This suggests that the effects of amiodarone on liver damage and the urea cycle might persist over time. Although the influence of other factors such as constipation, hypozincemia, and oral levetiracetam cannot be completely ruled out, this is the first case report showing several years of data on hyperammonemia due to amiodarone-induced liver injury in a Japanese case, and further studies are expected.

Finally, we present one sign of amiodarone exposure. Two iodine atoms exist in one amiodarone atom; in other words, 37 mg of iodine exists in 100 mg of amiodarone. Iodine in amiodarone and its metabolites remain in the liver, and this causes diffusely increased attenuation of the liver parenchyma [3]. By identifying the high density of the liver parenchyma on an abdominal CT scan, the clinician may be able to recognize the association between amiodarone and liver injury. Monitoring of liver function is crucial during amiodarone administration to avoid serious liver damage and encephalopathy. Close monitoring of liver enzymes and evaluation of liver CT imaging are recommended; in some cases, a liver biopsy should be considered. Clinicians should take great care of the patients who are using amiodarone and check for adverse effects.

## Conclusions

We report a case of chronic liver damage and urea cycle abnormalities due to long-term amiodarone use. Amiodarone accumulates in hepatocytes and affects the pathway of β-oxidation, which leads to apoptosis and necrosis of hepatic cells and causes steatohepatitis-like liver damage. Amiodarone impairs mitochondrial respiratory chain activity, and the decrease in acetyl-CoA reduces the availability of N-acetyl glutamic acid, which results in hyperammonemia.
